# Progress in targeting tumor-associated macrophages in cancer immunotherapy

**DOI:** 10.3389/fimmu.2025.1658795

**Published:** 2025-08-27

**Authors:** Wanqiu Xia, Xianghan Zhang, Yaru Wang, Zihan Huang, Xinyu Guo, Lei Fang

**Affiliations:** ^1^ Department of Gynaecology, The Second Affiliated Hospital of Harbin Medical University, Harbin, China; ^2^ Capital Medical University, Beijing, China; ^3^ Department of General Surgery, The Fourth Affiliated Hospital of Harbin Medical University, Harbin, China; ^4^ Key Laboratory of Preservation of Human Genetic Resources and Disease Control in China, Harbin Medical University, Ministry of Education, Harbin, China

**Keywords:** Tumor-associated macrophages, tumor microenvironment, immunosuppression, immune checkpoints, treatment resistance, immunotherapy

## Abstract

Tumor-associated macrophages (TAMs) are central to tumor progression, immune suppression, and resistance to therapy, making them promising targets in cancer immunotherapy. TAMs exhibit functional heterogeneity, polarizing into pro-tumor (M2-like) and anti-tumor (M1-like) phenotypes under different microenvironmental cues. M2-like TAMs promote immune evasion, angiogenesis, and metastasis, while M1-like TAMs enhance antitumor immunity. Combining TAM-targeted therapies with immune checkpoint inhibitors is also emerging as a potential strategy to enhance immunotherapy efficacy. This review outlines TAM-mediated immunosuppression mechanisms, including the secretion of VEGF, TGF-β, and immune checkpoint molecules like PD-L1. We also summarize the current strategies targeting TAMs, such as blocking the CD47/SIRPα axis, using CD40 agonists, and PI3Kγ inhibitors, which have shown promise in preclinical studies. Overall, this review underscores TAMs as pivotal therapeutic targets and proposes future directions to optimize combinatorial immunotherapy for enhanced clinical outcomes.

## 1 Introduction

Recent advancements in cancer immunotherapy have led to significant breakthroughs across various malignancies (1, 2). The core of these therapies involves reactivating innate and adaptive immune responses to induce robust antitumor immunity. Among these strategies, immune checkpoint inhibitors (ICIs), particularly monoclonal antibodies targeting programmed cell death-1 (PD-1)/programmed cell death-ligand 1 (PD-L1) and cytotoxic T lymphocyte-associated antigen-4 (CTLA-4), have shown notable therapeutic efficacy in multiple solid tumors (3–5). Despite their success, ICI monotherapy benefits only a subset of patients, with substantial intertumoral and interindividual variability in treatment outcomes (6). Moreover, many initially responsive patients develop acquired resistance to ICIs during treatment (7), underscoring the need for deeper insights into tumor progression mechanisms and novel therapeutic targets to improve immunotherapy efficacy.

The tumor microenvironment (TME) is a complex, immunosuppressive landscape comprised of diverse tumor cells, infiltrating immune cells, and stromal components (8–10). A growing body of evidence highlights that the immunosuppressive characteristics of the TME present a major obstacle to the success of immunotherapeutic strategies (11, 12). The TME is enriched with various immunosuppressive cell subsets, such as tumor-associated macrophages (TAMs) and regulatory T (Treg) cells. Notably, TAMs are the most abundant immune cell population within the TME (13). Emerging evidence underscores TAMs as central players in tumor angiogenesis, metastasis, immune evasion, and therapeutic resistance (14, 15). Importantly, targeting TAMs therapeutically has been shown to reduce resistance to ICIs (16). Both preclinical and clinical studies have demonstrated that combining TAM-targeted therapies with immune checkpoint blockade enhances antitumor efficacy (17). These findings position TAMs as a promising target for cancer immunotherapy. This review will discuss recent developments in TAM-targeted strategies and the current limitations of this approach.

## 2 Heterogeneity and plasticity of TAMs

Macrophages demonstrate remarkable plasticity and functional heterogeneity, assuming divergent roles within the TME based on external cues (18–21). Traditionally, they are dichotomized into M1 and M2 phenotypes: M1 macrophages, activated by IFN-γ, LPS, or TNF-α, exhibit tumoricidal and pro-inflammatory activity via secretion of IL-1β, IL-12, IL-23, and reactive nitrogen species; M2 macrophages, induced by IL-4, IL-10, or glucocorticoids, secrete IL-10 and TGF-β and express Arg1 and CD206, facilitating tissue repair, angiogenesis, and tumor progression (22). However, this binary model is now considered overly reductive, as macrophages often co-express M1 and M2 markers along a transcriptional continuum (18). IL−4/STAT6 signaling is a central driver of M2-like phenotypes: IL−4 binding to its receptor activates Janus kinases (JAKs), which phosphorylate STAT6, enabling STAT6 to translocate into the nucleus and induce transcription of anti-inflammatory and pro-tumor genes such as Arg1, MRC1 (CD206), and CCL18. Conversely, the NF−κB pathway, activated by stimuli such as LPS or TNF-α, is a hallmark of M1 polarization. Nuclear translocation of NF−κB subunits (p65/p50) induces transcription of pro-inflammatory cytokines (IL−1β, IL−12, TNF-α) that sustain anti-tumor immunity. Additionally, in the hypoxic tumor microenvironment, HIF-1α and HIF-2α stabilize and interact with co-activators to preferentially drive the expression of VEGF, CXCL12, and other genes that skew macrophages toward an immunosuppressive M2-like state. These signaling axes dynamically shape the transcriptional landscape of TAMs and contribute to their phenotypic plasticity within tumors.

Macrophage polarization is regulated by key transcription factors. M1 macrophages are driven by IRF5 and STAT1 signaling, while M2 macrophages are regulated by IRF3, IRF4, and STAT6, which promote anti-inflammatory gene transcription including Arg1 and IL-10 (23). Thus, reprogramming macrophage polarization holds therapeutic potential. In tumors, TAMs exhibit dynamic polarization. M1-like TAMs mediate anti-tumor effects through phagocytosis, ADCC, production of ROS and NO, and secretion of IFN-γ and IL-12 to enhance NK and CTL activity (24–26). Conversely, M2-like TAMs promote tumorigenesis by supporting angiogenesis, EMT, ECM remodeling, and immune suppression. They secrete VEGF, PDGF, EGF, FGF, TGF-β, MMPs, and cathepsins, and inhibit immunity via PD-L1, CD47/SIRPα, and cytokines like IL-10 and CCL2 (25–28). TAM heterogeneity contributes to variable clinical outcomes. Elevated TAM density is associated with poor prognosis in over 80% of cancers (11), particularly in lung and breast cancers, where M2-like TAMs associate with reduced survival (29). In breast cancer, stromal TAMs are more predictive of poor outcomes than intratumoral TAMs (30). Similarly, high TAM infiltration correlates with aggressive phenotypes in gastric, bladder, and skin cancers, multiple myeloma, and Hodgkin lymphoma (29). However, in colorectal cancer, TAMs may exert anti-tumor effects, correlating with CD8^+^ T cell infiltration and fewer metastases. Still, CD163^+^ TAMs in colorectal tumors predict poor outcomes (31). Collectively, the evidence supports the use of TAM infiltration levels as a predictive biomarker for patient prognosis in many solid tumors. Evaluating specific TAM subsets may offer more accurate prognostic insights in clinical oncology.

## 3 Pro-tumorigenic mechanisms of TAMs

### 3.1 Hypoxic microenvironment induces M2 polarization of TAMs

Owing to the rapid proliferation and expansion of tumor tissues, the TME often becomes hypoxic (32). Upon recruitment to the tumor site, macrophages are subjected to this hypoxic milieu, which activates multiple intracellular signaling pathways, including the hypoxia-inducible factor (HIF) pathway, the VEGF pathway, and the NF-κB pathway (33–35). These signaling cascades promote the accumulation of cytokines such as VEGF and eotaxin within tumor tissues, subsequently driving the polarization of macrophages toward the immunosuppressive M2 phenotype (36). M2-TAMs further secrete chemotactic factors including CCL2, CCL5, and macrophage colony-stimulating factor-1 (CSF-1), thereby contributing to immunosuppression and establishing a supportive niche for tumor angiogenesis, metastasis, and invasion (37, 38). M2-polarized macrophages actively reshape the TME by releasing IL−10 and TGF−β (39). IL−10 suppresses antigen presentation by dendritic cells and macrophages, downregulates MHC-II and costimulatory molecules, and inhibits cytotoxic T lymphocyte (CTL) activity, leading to an immune-permissive environment (40). TGF−β exerts pleiotropic effects by inducing epithelial–mesenchymal transition (EMT) in tumor cells, activating cancer-associated fibroblasts, and stimulating extracellular matrix deposition, all of which promote local fibrosis, tumor invasion, and immune exclusion (41). Importantly, the hypoxic TME also drives upregulation of biomarkers such as PD-L1 on circulating tumor cells (CTCs), which represents a mechanism of adaptive immune resistance; PD-L1-positive CTCs interact with PD-1 on T cells to dampen immune surveillance and are now being explored as predictors of ICI response (42, 43).

### 3.2 TAM-mediated angiogenesis

To accommodate the increased metabolic and oxygen demands associated with the high proliferation rate of tumor cells, TAMs undergo functional adaptation to promote angiogenesis and support tumor growth (44). The imbalance between pro-angiogenic and anti-angiogenic factors in the hypoxic tumor milieu leads to aberrant neovascularization, resulting in vasculature that is typically abnormal, immature, and highly permeable compared to normal blood vessels (45). Angiogenesis within tumors is a coordinated process involving both malignant and stromal cells and requires degradation of the basement membrane along with endothelial cell proliferation and migration (46). TAMs actively participate in this process by secreting matrix metalloproteinases (MMPs) and cathepsins that degrade extracellular matrix components (47). Furthermore, they produce key pro-angiogenic factors such as VEGF, platelet-derived growth factor (PDGF), basic fibroblast growth factor (b-FGF), and chemokines including CCL2 and CXCL8, which collectively facilitate the formation of a vascular network essential for sustained tumor expansion and dissemination (48).

VEGF plays a central mechanistic role: it binds VEGFR2 on endothelial cells, stimulating their proliferation, migration, and survival, while also increasing vascular permeability (49, 50). VEGF also indirectly suppresses anti-tumor immunity by impairing dendritic cell maturation and promoting anergic or exhausted T cell phenotypes, thereby coupling angiogenesis with immune evasion (51). Additionally, TGF−β released by M2 macrophages augments angiogenesis through induction of extracellular matrix remodeling, fibroblast activation, and production of angiogenic ligands, while IL−10 reduces inflammatory cues that might otherwise restrain angiogenesis (52, 53). Together, these factors foster a structurally and functionally aberrant vascular network that facilitates tumor perfusion and dissemination. Notably, factors such as VEGF-A and CCL2 can recruit circulating monocytes, and their expression levels positively correlate with TAM accumulation and vascular density in certain tumor types (44, 54). Of particular interest is a monocyte subpopulation characterized by the expression of the tyrosine kinase receptor Tie2 which has gained increasing attention for its role in promoting angiogenesis (55, 56). Biel et al. demonstrated that angiopoietin-1 (Ang-1), the ligand of Tie2, is expressed by endothelial cells and promotes perivascular alignment of TEMs. These cells subsequently secrete Wnt-7b, which targets endothelial cells and induces VEGF production, thus enhancing angiogenesis (57–60). Collectively, TAMs act in concert with tumor-derived angiogenic factors to facilitate neovascularization, laying the groundwork for tumor proliferation and progression.

### 3.3 TAMs and tumor metastasis

**Figure 1:**
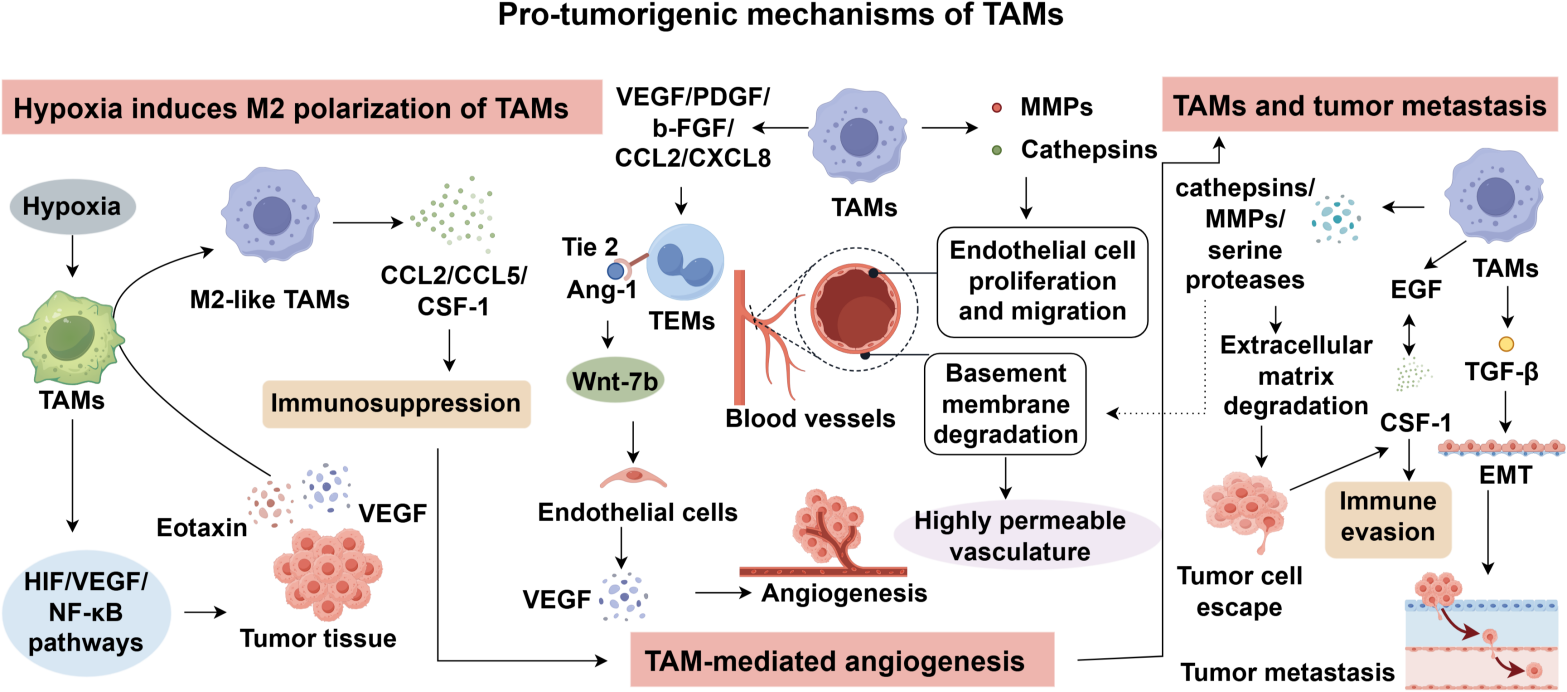
Pro-tumorigenic mechanisms of TAMs.

Tumor invasion and metastasis represent the leading cause of cancer-related mortality (61, 62). Substantial evidence supports a functional association between TAM recruitment and tumor cell dissemination. TAMs participate in the formation of the pre-metastatic niche, thereby fostering colonization at distant sites. Moreover, within the metastatic microenvironment, TAMs facilitate tumor cell extravasation and survival by mediating immune evasion (63). Epithelial-to-mesenchymal transition (EMT) is a critical step in the metastatic cascade, whereby epithelial tumor cells acquire migratory and invasive properties, enabling survival and dissemination via hematogenous or lymphatic routes. TAMs have been implicated in the regulation of this process (64). For instance, TGF-β secreted by TAMs can induce EMT in tumor cells. In teratomas, TAM accumulation is associated with elevated TGF-β expression, which in turn triggers EMT and promotes metastasis (65). Additionally, TAMs secrete proteolytic enzymes such as cathepsins, MMPs, and serine proteases, which degrade basement membranes and extracellular matrix, facilitating tumor cell escape from the primary site. Furthermore, tumor-derived CSF-1 interacts with epidermal growth factor (EGF) signaling in macrophages, promoting their perivascular accumulation and enabling immune evasion by tumor cells (54). These findings suggest that TAM accumulation significantly enhances tumor cell invasiveness and metastatic potential. Therefore, targeting TAMs may offer a promising strategy to counteract metastasis and enhance the efficacy of cancer immunotherapy (**Figure 1**).

## 4 Advances in targeting TAMs for cancer therapy

### 4.1 Inhibition of pro-tumor TAMs

#### 4.1.1 Blocking monocyte/macrophage recruitment

Inhibiting monocyte/macrophage recruitment to the TME limits pro-tumor TAM accumulation. CCL2–CCR2 and CXCL12–CXCR4 axes are key mediators of this process (66). CCL2, secreted by tumor and stromal cells, recruits CCR2^+^ monocytes, promoting TAM differentiation; anti-CCL2 antibodies reduce TAM infiltration and tumor progression (67). However, CCL2 blockade withdrawal accelerated metastasis in breast cancer models, likely due to reactive monocytosis (68). Phase I trials of CCL2/CCR2 inhibitors showed modest benefit, while Phase II lacked efficacy, possibly due to compensatory CCL2 upregulation (69). Notably, CCR2 antagonists plus chemotherapy showed improved outcomes in pancreatic cancer compared to chemotherapy alone (70). CXCL12 recruits immunosuppressive TAMs and impairs T cell activation; CXCR4 blockade reduces TAM chemotaxis and delays tumor growth in preclinical models (71, 72). Similar effects were seen in ovarian and prostate cancers (25). Additionally, the CX3CL1/CX3CR1 axis promotes TAM-driven skin carcinogenesis (73) (**Table 1**).

**Table 1:** Therapeutic strategies targeting tumor-associated macrophages (TAMs) in cancer immunotherapy.

Strategy	Target molecule/Pathway	Mechanism of action	Preclinical/Clinical agents	Tumor types studied	Clinical Status
Inhibition of TAM Recruitment	CCL2–CCR2 axis	Blocks monocyte chemotaxis into tumor sites	Carlumab (anti-CCL2), PF-04136309 (CCR2 inhibitor)	Breast, pancreatic cancer	Phase I/II (limited efficacy)
	CXCL12–CXCR4 axis	Prevents immunosuppressive macrophage recruitment	AMD3100 (plerixafor), NOX-A12	Ovarian, prostate, pancreatic cancer	Phase I/II
Depletion of TAMs	CSF-1/CSF-1R axis	Induces TAM apoptosis and reduces M2 macrophage survival	PLX3397, Emactuzumab	Glioblastoma, breast cancer	Phase I/II
	Broad cytotoxic agents	Directly depletes macrophages	Trabectedin, Bisphosphonates (e.g., Zoledronate)	Soft tissue sarcoma, breast cancer	Approved (off-label use)
Targeting TAM-Derived Immunosuppression	IL-10, TGF-β, VEGF	Neutralizes immunosuppressive cytokines and pro-angiogenic factors	Anti-IL-10 antibody, TGF-β inhibitors, Bevacizumab	Colorectal, liver, breast cancer	Approved/clinical trials
	Complement (C3aR, C5aR1)	Inhibits complement-mediated TAM recruitment and polarization	PMX53 (C5aR1 antagonist), anti-C1q antibody	Lung, breast, melanoma	Preclinical/early clinical
Immune Checkpoint Targeting on TAMs	PD-1/PD-L1	Restores phagocytosis and cytotoxicity of TAMs	Nivolumab, Atezolizumab	Multiple solid tumors	Approved
	TREM2, MARCO, CD206	Reprograms suppressive TAMs to M1-like phenotypes	Anti-TREM2 antibody, RP-182 (CD206 modulator)	Glioma, breast, melanoma	Preclinical
TAM Reprogramming	CD40 agonists	Converts M2-TAMs to M1 phenotype, promotes antigen presentation	Selicrelumab, CD40L-based therapies	Pancreatic, colorectal cancer	Phase I/II
	PI3Kγ inhibitors	Blocks M2 signaling and induces immune-stimulatory macrophage polarization	IPI-549, TG100-115	Pancreatic, breast cancer	Phase I
	HDAC (Class IIa) inhibitors	Enhances pro-inflammatory TAM gene expression	TMP195	Breast cancer	Preclinical
Macrophage-Based Cellular Therapy	CAR-Macrophages (CAR-M)	Engineered macrophages enhance phagocytosis and antigen presentation	HER2-targeted CAR-M, CTLA4-CAR-M	Breast, ovarian, pancreatic cancer	Phase I/Preclinical

#### 4.1.2 Targeting pro-tumor complement components

Beyond blocking recruitment, inducing TAM apoptosis offers another avenue to deplete these cells. The CSF-1/CSF-1R axis is critical for monocyte/macrophage differentiation, maturation, and survival. Inhibition of this pathway induces TAM death and attenuates their pro-tumor functions (14, 74). Agents such as trabectedin and bisphosphonates have been shown to eliminate macrophages through apoptosis. Trabectedin induces DNA damage and G2/M arrest in tumor cells, and exhibits anti-proliferative activity in melanoma (75). Bisphosphonates enhance immunosurveillance, inhibit tumor invasiveness, and reduce angiogenesis, while also synergizing with other anticancer agents (76). Both preclinical and clinical studies in breast cancer have validated the anti-tumor potential of bisphosphonates targeting TAMs (77). Recent evidence underscores the tumor-promoting role of complement in human and murine cancers. Activation via classical (C1q), alternative, or lectin pathways generates C3a/C5a and the membrane attack complex. C5a recruits MDSCs, enhancing immunosuppression, while C3a/C3aR signaling drives TAM recruitment and immune evasion. In squamous carcinoma, urokinase-positive macrophages mediate C5a release, promoting pro-tumor TAMs and suppressing T cell cytotoxicity (78). Prognostic complement markers include C4d, C5a/C5aR1, and C1s/C4d (79, 80). High expression of complement genes correlates with poor prognosis in melanoma, glioma, and ccRCC (79). C1q^+^ TAMs induce immunosuppression via PD-1, LAG-3, and PD-L2 (80). Complement inhibition synergizes with ICB; dual C5a or C3aR and PD-1 blockade enhances CD8^+^ T cells, reduces MDSCs, and improves survival (78).

#### 4.1.3 Immune checkpoints on TAMs

TAM subsets defined by surface markers, such as CD163^+^ and CD206^+^ macrophages, have distinct functional roles and are associated with different prognostic outcomes across multiple cancer types. Scavenger receptors on TAMs are promising targets for macrophage reprogramming. CD163, an M2 marker, is strongly linked to immunosuppressive functions and poor prognosis in cancers such as pancreatic cancer and melanoma (25) Depletion or functional blockade of CD163^+^ TAMs has been shown to enhance T-cell–mediated immunity and improve responses to PD−1 blockade in preclinical studies (81). Similarly, CD206 defines another immunosuppressive TAM subset that secretes high levels of IL−10 and promotes tumor immune evasion. RP-182, a synthetic peptide targeting CD206, eliminates CD206^+^ cells and reprograms TAMs into M1-like macrophages, enhancing phagocytosis and antitumor activity, with synergistic effects in immunotherapy models (25). Given the distinct prognostic implications of CD163^+^ and CD206^+^ subsets, specifically targeting these populations has emerged as a precision strategy to improve clinical outcomes. MARCO, enriched in glioblastoma TAMs, also supports immunosuppression; its blockade reprograms TAMs (14). TAMs expressing PD-1 show reduced phagocytosis, and tumor PD-L1 impairs both T cell and macrophage functions; PD-1/PD-L1 blockade restores immunity (82). TREM2, upregulated in TAMs across cancers, limits PD-1 blockade efficacy; anti-TREM2 antibodies are under clinical evaluation (25, 83). These findings highlight that focusing on specific immunosuppressive TAM subsets, such as CD163^+^, CD206^+^, MARCO^+^, or TREM2^+^ macrophages, may enable more precise interventions and better therapeutic efficacy in combination with immune checkpoint inhibitors.

### 4.2 Activation of anti-tumoral TAMs

While depletion of TAMs has demonstrated antitumor potential, the tumoricidal capacity of macrophages also merits attention. Besides the protumoral, immunosuppressive TAM phenotype dominant in the TME, antitumoral macrophages exist. As complete depletion may lead to chronic inflammation or infection, reprogramming TAMs into antitumor phenotypes is a promising alternative (14, 84). Myeloid cells, including macrophages, express SIRPα, which binds CD47 to inhibit phagocytosis (25) Thus, the CD47/SIRPα axis represents a target for immunotherapy. Blocking this axis restores macrophage phagocytosis and elicits antitumor immunity (85). Anti-CD47 antibodies suppress tumor growth, enhance antitumoral macrophage recruitment, and activate CD8^+^ T cells in multiple tumor models, including glioblastoma, where CD47 blockade reprogrammed TAMs (25, 85). Anti-CD47 therapy also synergizes with immune checkpoint blockade, amplifying efficacy and reducing metastasis (85). Other TAM-modulating agents include CD40 agonists, PI3Kγ inhibitors, and class IIa histone deacetylase (HDAC) inhibitors. CD40, a TNF receptor family member expressed on tumor cells and antigen-presenting cells, including macrophages, induces proinflammatory cytokine release and upregulation of CD80 and CD86 upon activation, sustaining T cell responses (29, 86). In pancreatic ductal adenocarcinoma, CD40 activation converted immunosuppressive TAMs to immunostimulatory ones, restoring immune surveillance (29). In murine colon cancer, CD40 agonists combined with CSF-1R inhibitors reduced immunosuppressive cells and increased antitumoral TAMs (33), and also showed synergy with checkpoint blockade and chemotherapy (86). Thus, CD40 activation facilitates macrophage repolarization and enhances immunotherapy response (29).

PI3Kγ is highly expressed in myeloid cells and promotes immunosuppression via NF-κB inhibition (86, 87). PI3Kγ blockade reactivates T cell responses and inhibits tumor progression (87). In breast and pancreatic cancer models, PI3Kγ deletion or inhibition reprogrammed TAMs, alleviated immunosuppression, and reduced tumor invasion and metastasis (86). Moreover, PI3Kγ inhibitors synergized with checkpoint blockade to improve tumor control and prognosis *in vivo* (86). HDACs regulate gene expression by removing acetyl groups from histone and non-histone proteins (88). TMP195, a selective class IIa HDAC inhibitor, promoted recruitment and differentiation of phagocytic, proinflammatory macrophages in the TME, reprogramming TAMs and reducing tumor burden and metastasis. In murine breast cancer models, TMP195 also enhanced tumor killing when combined with chemotherapy or checkpoint inhibitors (89). Overall, reprogramming TAMs into tumoricidal phenotypes offers substantial therapeutic promise and a novel avenue for cancer immunotherapy.

### 4.3 Macrophage-based therapy

In recent years, chimeric antigen receptor T cell (CAR-T) therapy has achieved remarkable success in the treatment of hematologic malignancies. However, its efficacy in solid tumors remains limited, primarily due to tumor heterogeneity and the profoundly immunosuppressive tumor microenvironment (90). As an alternative, chimeric antigen receptor macrophages (CAR-M) have emerged as a novel form of cell-based immunotherapy designed to harness the innate phagocytosis, cytokine release, activation of the tumor microenvironment, and antigen-presenting capacity of macrophages. Unlike CAR-T cells, CAR-M can infiltrate solid tumors more effectively and remodel the immunosuppressive milieu through secretion of proinflammatory cytokines, enhanced phagocytosis, and cross-priming of tumor-specific T cells (91). Currently, several clinical trials are underway or in development to evaluate the therapeutic potential of CAR-M across different malignancies. Phase 1 clinical trial of CT-0508 has shown that HER2-targeted CAR-M therapy achieves significant antitumor responses in murine tumor models (NCT04660929) (92). This approach not only mediates direct tumor cell killing but also promotes the phenotypic shift from M2- to M1-type macrophages, thereby amplifying T cell-mediated antitumor responses. However, several limitations remain to be addressed. Unlike T cells, macrophages have limited proliferative and expansion capacity, which may significantly constrain the overall therapeutic efficacy. Additionally, excessive macrophage activation may lead to overproduction of proinflammatory cytokines, resulting in potential cytotoxicity (91). Despite these challenges, CAR-M therapy represents an exciting frontier in solid tumor immunotherapy, and continued investigation and optimization are warranted.

## 5 Conclusion

TAMs play pivotal roles in tumor progression, immune suppression, and therapeutic resistance, orchestrating various aspects of tumor biology, including angiogenesis, metastasis, and immune evasion. Their functional heterogeneity and plasticity enable TAMs to adopt pro-tumor (M2-like) or anti-tumor (M1-like) phenotypes, contributing to the complex TME. While M1-like TAMs are associated with anti-tumor immunity, M2-like TAMs support tumor progression by promoting angiogenesis, immune suppression, and metastasis. These findings underscore the potential of targeting TAMs in cancer immunotherapy, with strategies like macrophage reprogramming, recruitment inhibition, and immune checkpoint blockade showing promise in preclinical and early-phase clinical trials.

However, several challenges remain in TAM-targeted therapies. The dynamic heterogeneity of TAM subsets necessitates the identification of precise biomarkers to guide treatment selection. Additionally, compensatory mechanisms and the impact of standard therapies on TAM function require further investigation. Future research should focus on understanding TAM diversity through spatial multi-omics, optimizing combination regimens with immune checkpoint inhibitors (ICIs), and exploring novel targets such as complement cascades (C3aR/C5aR) and scavenger receptors (TREM2). By addressing these gaps, TAM-targeted strategies may significantly enhance immunotherapy outcomes and expand their clinical applicability across various cancers.
